# Use of the Clock Drawing Test and the Rey–Osterrieth Complex Figure Test-copy with convolutional neural networks to predict cognitive impairment

**DOI:** 10.1186/s13195-021-00821-8

**Published:** 2021-04-20

**Authors:** Young Chul Youn, Jung-Min Pyun, Nayoung Ryu, Min Jae Baek, Jae-Won Jang, Young Ho Park, Suk-Won Ahn, Hae-Won Shin, Kwang-Yeol Park, Sang Yun Kim

**Affiliations:** 1 Department of Neurology, College of Medicine, Chung-Ang University, Seoul, Republic of Korea; 2 Department of Medical Informatics, Chung-Ang University College of Medicine, Seoul, Republic of Korea; 3 Department of Neurology, Seoul National University College of Medicine & Seoul National University Bundang Hospital, Seoul, Republic of Korea; 4 Department of Neurology, Kangwon National University Hospital, Kangwon National University College of Medicine, Chuncheon, Republic of Korea; 5 Department of Neurology, Seoul National University College of Medicine & Neurocognitive Behavior Center, Seoul National University Bundang Hospital, 300 Gumi-dong, Bundang-gu, Seongnam-si, Gyeonggi-do 463-707 Republic of Korea

**Keywords:** Clock Drawing Test, Cognitive impairment, Convolutional neural network, Machine learning, Rey–Osterrieth Complex Figure Test, TensorFlow

## Abstract

**Background:**

The Clock Drawing Test (CDT) and Rey–Osterrieth Complex Figure Test (RCFT) are widely used as a part of neuropsychological test batteries to assess cognitive function. Our objective was to confirm the prediction accuracies of the RCFT-copy and CDT for cognitive impairment (CI) using convolutional neural network algorithms as a screening tool.

**Methods:**

The CDT and RCFT-copy data were obtained from patients aged 60–80 years who had more than 6 years of education. In total, 747 CDT and 980 RCFT-copy figures were utilized. Convolutional neural network algorithms using TensorFlow (ver. 2.3.0) on the Colab cloud platform (www.colab.research.google.com) were used for preprocessing and modeling. We measured the prediction accuracy of each drawing test 10 times using this dataset with the following classes: normal cognition (NC) vs. mildly impaired cognition (MI), NC vs. severely impaired cognition (SI), and NC vs. CI (MI + SI).

**Results:**

The accuracy of the CDT was better for differentiating MI (CDT, 78.04 ± 2.75; RCFT-copy, not being trained) and SI from NC (CDT, 91.45 ± 0.83; RCFT-copy, 90.27 ± 1.52); however, the RCFT-copy was better at predicting CI (CDT, 77.37 ± 1.77; RCFT, 83.52 ± 1.41). The accuracy for a 3-way classification (NC vs. MI vs. SI) was approximately 71% for both tests; no significant difference was found between them.

**Conclusions:**

The two drawing tests showed good performance for predicting severe impairment of cognition; however, a drawing test alone is not enough to predict overall CI. There are some limitations to our study: the sample size was small, all the participants did not perform both the CDT and RCFT-copy, and only the copy condition of the RCFT was used. Algorithms involving memory performance and longitudinal changes are worth future exploration. These results may contribute to improved home-based healthcare delivery.

**Supplementary Information:**

The online version contains supplementary material available at 10.1186/s13195-021-00821-8.

## Background

There is a growing interest in the use of artificial intelligence in clinical practice [1–3]. Efforts are underway for the prediction and diagnosis of prodromal or early-stage dementia [4–7] at home and in clinical settings [8, 9].

Performing a cognitive assessment is essential for establishing an objective diagnosis in patients with cognitive complaints [10]. Most of the currently used screening tools have been constructed based on neuropsychological tests. The Rey–Osterrieth Complex Figure Test (RCFT) is widely used by neuropsychologists to assess cognitive function. The test was first developed by Rey in 1941 [11] and has proved to be a useful tool for analyzing visuospatial construction, perceptual organization, and visual memory in clinical evaluations and research studies [12]. Patients with parieto-occipital lesions, especially on the right side, have difficulties in spatial organization while drawing, probably because of visual disorientation [13]. Patients with frontal lobe damage show impairment in programming abilities with respect to figure reproduction [14, 15]. Patients with early-stage Alzheimer’s disease (AD) perform poorly on this test [16]. Seo et al. showed that that the copy condition of the test was associated with spatial organization and planning, and it significantly predicted the conversion to pre-MCI or MCI [17]. The salience of visuospatial and organizational skills as evaluated by the copy condition of the RCFT differs according to the level of intelligence [18]. To obtain a more quantitative value for the accuracy of a participant’s drawing, many researchers use the RCFT based on the Osterrieth scoring criteria to diagnose cognitive impairment (CI) [19].

The Clock Drawing Test (CDT) is also widely used as a screening test for patients with dementia because it is easy to use and reflects a variety of cognitive functions, including visuospatial function, frontal lobe execution, and memory (of clock-related concepts). The CDT requires a participant to draw the hour and minute hands of the clock to show the time “11:10”. In patients with frontal lobe dysfunction, abstract thinking is compromised, which makes them prone to stimulus-bound errors wherein they process information at a more perceptual level than at a semantic level. Thus, they have difficulty recording “10” as “2”, and since “10” is adjacent to “11”, their attention is pulled toward the “10”, and they set the minute hand to “10” instead of “2” [20, 21]. Studies related to dementia have reported that the CDT is useful in the screening of cognitive impairment [22, 23] and that it can be used for screening MCI [11]. The CDT has a variety of scoring systems [24]. Among them, the Consortium to Establish a Registry for Alzheimer’s Disease CDT [25] is known to be the simplest method with a high diagnostic efficiency [26].

Detecting the severity of dementia is important for clinical and research purposes, and the Clinical Dementia Rating Scale (CDR) is one of the most commonly used tools for this assessment. The CDR comprises the global and sum of boxes (SOB) scores. The CDR-SOB score is considered a more detailed quantitative index than the global score and provides more information regarding patients with mild dementia. Previous studies have shown that the CDR-SOB scores may have the potential for discriminating between patients with MCI and those with very early stage AD dementia who are assigned a global CDR score of 0.5. Patients with MCI were assigned a CDR-SOB score of 1.8 ± 0.8, and those with very mild AD were assigned a CDR-SOB score of 3.0 ± 0.8 [27]. O’Bryant et al. classified the severity of dementia (normal to severe) based on SOB scores (0–18). In their system, a CDR-SOB score of 0 indicated normal cognition, 0.5–2.5 indicated suspicious damage, and 3.0–4.0, indicated very mild dementia [28]. Our database did not contain clinical information such as that regarding MCI or dementia, which is why the participants in this study were classified as having normal, mild, and severe CI based on the CDR-SOB score. However, unlike O’Bryant et al., we arbitrarily classified the degree of cognitive impairment: normal cognition (NC), 0–1.5; mild impairment of cognition (MI), 2.0–3.5; and severe impairment of cognition (SI), 4-.

Several studies have demonstrated that a digital CDT of a limited number of participants was able to differentiate patients with AD and other dementia syndromes from healthy controls using machine learning [29, 30]. However, digital CDT requires special equipment, and in deep learning, a greater amount of data with a good quality yields better result. Therefore, we predicted CI with deep learning based on a greater amount of drawing test data than that analyzed in previous studies. We investigated whether the CDT and RCFT can be used as screening tests to predict CI using convolutional neural network (CNN) algorithms. We also investigated whether the CDT, which measures various cognitive functions, was better than the RCFT in predicting CI. Our objective was to evaluate the prediction accuracies of these two tests for CI and compare them.

## Methods

### Dataset

Anonymous neuropsychological data from Jan. 2018 to Sep. 2020 at the Memory Clinics at Seoul National University Bundang Hospital and Chung-Ang University Hospital were retrospectively collected. The RCFT-copy and CDT figures that were drawn by patients aged 60–80 years with more than 6 years of education were selected. There were a total of 747 CDT and 980 RCFT-copy figures.

The original RCFT [11] and CDT were conducted by trained psychologists in the neurology outpatient testing room. The participants were given an A4 size paper and a pencil and instructed to copy the “Rey complex figure” and/or draw a “clock” indicating the time “11:10.” During the CDT, the following instructions were given: “You have to draw a clock. Draw a circle first and write all the numbers in it.” After the patients wrote the numbers, they were instructed as follows: “Now draw hands on the clock to indicate the time 11:10.” Test participants were clinically classified by dementia-specialized clinicians based on the CDR-SOB score into the following groups: normal cognition (NC), 0–1.5; mild impairment of cognition (MI), 2.0–3.5; and severe impairment of cognition (SI), 4- [28].

### Model training and statistical analyses

The datasets of the CDT and RCFT-copy figures were organized into four classes: NC vs. MI, NC vs. SI, NC vs. CI (MI+SI), and NC vs. MI vs. SI. The datasets were prepared for three 2-way evaluations and one 3-way evaluation with respect to each CDT and RCFT-copy figure. The 2-way classifications for differentiating MI, SI, or CI from NC were performed in the CDT and RCFT datasets. The 3-way classification differentiated NC, MI, and SI in both the datasets.

All the algorithms were performed on the Colab cloud platform (www.colab.research.google.com). To model each algorithm, the dataset was subjected to the following preprocessing steps. As the dataset was relatively small number for machine learning, we augmented the image data. We made a replica image with a 10% height reduction and another one with a 10% width reduction, compared to each original drawing. All the images including the original drawing and two replicas were placed in a 600-dpi template.

TensorFlow (ver. 2.3.0) on Colab, which is a commonly used open-source, Python-based software library for machine learning developed by Google was used for preprocessing and modeling [31]. As an example, the code that predicted CI in the RCFT-copy dataset is given in the Supplementary Table. We imported the data in the “.png” format and used the “validation_split” function from “tf.keras.preprocessing.image_dataset_from_directory” to randomly split the data into training and test datasets. The training data size was 70%, which indicated the percentage of the data to be withheld for training; the validation dataset was thus composed of the remaining 30% of the data. The features were normalized with “tensorflow.keras.layers.experimental.preprocession.Rescaling(1./255).” We implemented data augmentation with “RandomZoom” and “RandomRotation” using “experimental Keras Preprocessing Layers.” This artificial neural network consists of five convolutional and maxpooling layers, and a dropout layer was inserted before connecting it to a fully connected neural network. The dropout rate was 0.2–0.3; therefore, 2–3 out of 10 weights were connected to the next layer to prevent overfitting. The cost was calculated using “Sparse_Categorical_Crossentropy” and minimized by means of the “adam” optimizer method. Model training was performed with a batch size of 20 and 40–72 epochs, depending on the dataset. During model training, optimal dropout rates and epochs were found and adjusted. After the dropout rate and epochs were defined, we obtained the average prediction accuracy based on 10 trials.

## Results

Demographic and clinical characteristics including age, education level, and Mini-Mental Status Examination scores of the CDT and RCFT-copy datasets are given in Table 1. No differences in age and education levels were found among the NC, MI, and SI groups in the CDT and RCFT-copy datasets (*p* > 0.05).

**Table 1:** Demographic and clinical characteristics according to cognition status based on the CDT and RCFT-copy

		NC	MI	SI	Total
CDT	No. of participants	454 (60.8%)	179 (24.0%)	114 (15.3%)	747
	Age (years ± *SD*)	69.1 ± 7.6	70.5 ± 7.7	72.8 ± 7.5	
	Education (years ± *SD*)	11.6 ± 3.2	11.2 ± 3.9	10.5 ± 4.2	
	MMSE	26.0 ± 3.1	24.4 ± 2.3	21.9 ± 2.9	
RCFT-copy	No. of participants	411 (41.9%)	367 (37.4%)	202 (20.6%)	980
	Age (years ± *SD*)	70.2 ± 7.8	70.2 ± 7.2	72.7 ± 6.1	
	Education (years ± *SD*)	11.9 ± 3.5	11.2 ± 3.7	10.5 ± 3.4	
	MMSE	26.2 ± 3.2	24.5 ± 2.4	21.7 ± 2.8	

*CDT*, clock drawing test; *RCFT*, Rey–Osterrieth Complex Figure Test; *NC*, normal cognition; *MI*, mild impairment of cognition; *SI*, severe impairment of cognition

**Fig. 1:**
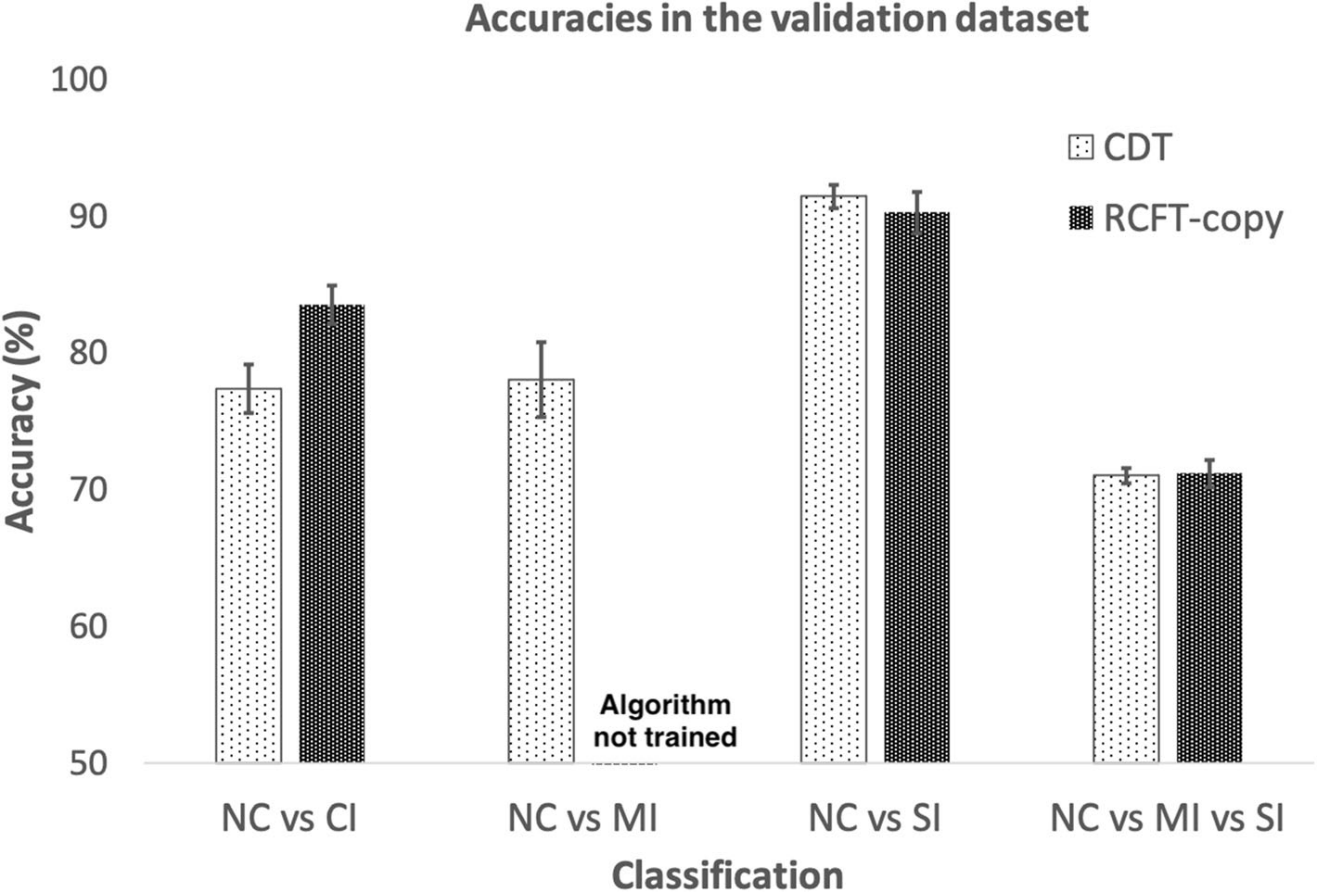
Accuracies of algorithms for the prediction of cognitive impairment in the validation datasets of CDT and RCFT-copy figures. CDT, clock drawing test; RCFT-copy, Rey–Osterrieth Complex Figure Test-copy

The CDT was more accurate in differentiating MI (CDT, 78.04 ± 2.75%; RCFT, not being trained) and SI from NC (CDT, 91.45 ± 0.83%; RCFT, 90.27 ± 1.52%) (Table 2); however, the RCFT-copy was better at predicting CI (CDT, 77.37 ± 1.77%; RCFT, 83.52 ± 1.41%). The accuracy of the 3-way classification (NC vs. MI vs. SI) was approximately 71% (Fig. 1).

**Table 2:** Mean accuracies of the CDT and RCFT-copy for the prediction of cognitive impairment

	No. of images	No. of images training: validation dataset	Mean accuracy of validation dataset (%)
CDT	NC vs CI (1362:879)	1569:672	77.37 ± 1.77
	NC vs MI (1362:537)	1330:569	78.04 ± 2.75
	NC vs SI (1362:342)	1193:511	91.45 ± 0.83
	NC vs MI vs SI (1362:537:342)	1569:672	71.06 ± 0.75
RCFT-copy	NC vs CI (1233:1707))	2058:882	83.52 ± 1.41
	NC vs MI (1233:1101)	1634:700	NT
	NC vs SI (1233:606)	1288:551	90.10 ± 1.33
	NC vs MI vs SI (1233:1101:606)	2058:882	71.50 ± 1.16

*CDT*, clock drawing test; *RCFT*, Rey–Osterrieth Complex Figure Test; *NC*, normal cognition; *MI*, mild impairment of cognition; *SI*, severe impairment of cognition; *NT*, not being trained

## Discussion

The algorithm for predicting CI was more accurate in the RCFT-copy dataset than in the CDT dataset, but the algorithm for predicting MI in the RCFT-copy dataset had not being trained. However, at other levels of cognitive impairment, namely the prediction of SI in a 2-way classification and MI and SI in a 3-way classification, the two tests were nearly equal in their predictive accuracy in both the CDT and RCFT-copy datasets. We had expected the CDT to be superior to the RCFT-copy in predicting CI; this is because the RCFT-copy simply evaluates visual constructional function based on the copying of the figure, whereas the CDT not only evaluates visual constructional function and clock semantics but also the inhibitory function [20]. The CDT was thought to be more advantageous for evaluating various aspects of cognitive function. However, there was no difference between the two tests in distinguishing MI and SI from NC in the 3-way classification. Rather, the RCFT was better at distinguishing CI (including MI and SI) from NC in the 2-way classification. A voxel-based morphometric study that evaluated the relationship between the RCFT and brain volume showed that the RCFT score and the right caudate nucleus volume were positively correlated [32]. Therefore, in addition to visuospatial function evaluation, the RCFT may be used to evaluate frontal executive function. Another study found that poor copy scores in the RCFT were associated with greater beta amyloid burden in the frontal area on C-Pittsburgh B positron emission tomography/computed tomography and F-FC119S positron emission tomography/computed tomography [33]. These studies have shown that the RCFT is associated with cognitive functions other than the visual constructional function.

In our study, the prediction of SI in the validation dataset had an accuracy greater than 90%; however, the accuracy of differentiating between MI and NC was less than 80%. A slight cognitive decline was difficult to detect using the CDT. Moreover, the algorithm predicting MI in the RCFT-copy dataset was not well-trained. For differentiating MI from NC in a 2-way classification of the RCFT-copy dataset, the algorithms’ accuracy was only approximately ~ 55% and too variable; we did not consider this to be meaningful training, because a probability of approximately 50% exists even at random. However, it was better at predicting CI than the CDT algorithm was. Since prediction with RCFT-copy using the MI dataset did not show a good accuracy, it can be expected that the prediction of CI in a CI dataset that includes this MI dataset will not be accurate. Even if the MI prediction did not have a good accuracy, the algorithm using the CI dataset would produce different features with different weights, leading to better results than expected. However, we were unable to figure out what features the machine extracts and how much weight it gives to them.

There is a limitation in directly comparing the RCFT-copy and CDT machine learning algorithms as not all the participants performed both tests; only 91.7% (685/747) of the participants in the CDT dataset and 94.2% (923/980) of those in the RFCT-copy dataset had performed both tests. Overall, the two algorithms that predict CI seemed to have worked well. However, as the RCFT-copy algorithm could not be trained to select a patient with MI, the CDT algorithm seemed relatively advantageous.

Age and education have a strong effect on the performance in these tests [34, 35]. Most of the available norms provide either percentile scores/means and standard deviations for age-defined classes. However, in this study, age and educational levels were not included in the algorithm. We selected participants according to age and education levels; their age ranged from 60 to 80 years, as changes in cognitive function are expected in this age range, and they had more than 6 years of formal education (to minimize the impact of a low level of education on the performance in the drawing tests). If more substantial data could have been obtained, it would have been possible to predict CI based on the variables of age and education level.

We found that the machine learning algorithms based on the RCFT-copy and CDT datasets worked well in terms of predicting CI. Although the two drawing tests alone cannot sufficiently predict CI cross-sectionally, detecting changes in cognition using a longitudinal dataset is worth future exploration. It should be noted that the drawing test alone does not substitute for formal neuropsychological tests to predict overall CI. This study suggests the potential for home-based care services using drawing test algorithms to monitor or screen for CIs.

### Limitations

There are several limitations to our study. The small sample size may limit the generalizability of our findings. All the participants did not perform both the CDT and RCFT-copy, which can limit the direct comparison of the two tests. In this study, only the copy condition of the RCFT was used. The RCFT consists of the copy and visual memory recall (immediate and delayed recall) conditions; memory performance is important for the screening of CI. Therefore, including the delayed recall condition of the RCFT in future studies may help in better prediction of CI. The memory recall condition of the RCFT is thought to require a more complex machine learning model; this approach will be attempted in our future research.

## Conclusions

The CDT and RCFT-copy showed good performance for predicting SI; however, drawing tests alone are not enough to predict overall CI. Results from drawing tests and CNN algorithms can help improve home-based healthcare delivery. Algorithms involving memory performance and longitudinal changes are worth exploring in future studies.

## Supplementary Information

**Additional file 1: Supplementary Table**. The codes for prediction of the cognitive impairment with the two drawing test.

## Data Availability

The datasets used and/or analyzed during the current study are available from the corresponding author on reasonable request.

## Abbreviations

AD: Alzheimer’s disease
CDT: Clock drawing test
RCFT: Rey–Osterrieth Complex Figure Test
RCFT-copy: Rey–Osterrieth Complex Figure Test-copy
CNN: Convolutional neural network
MCI: Mild cognitive impairment
NC: Normal cognition
MI: Mild impairment of cognition
SI: Severe impairment of cognition
CI: Cognitive impairment
CDR: Clinical Dementia Rating Scale
SOB: Sum of Boxes
