# Supplementary material for: Use of the Clock Drawing Test and the Rey–Osterrieth Complex Figure Test-copy with convolutional neural networks to predict cognitive impairment
Source: Alzheimers Res Ther. 2021 Apr 20;13:85. doi: 10.1186/s13195-021-00821-8 (PMC8059231; doi:10.1186/s13195-021-00821-8)
Supplement: Supplementary file 1 — Additional file 1: Supplementary Table. The codes for prediction of the cognitive impairment with the two drawing test. [file 13195_2021_821_MOESM1_ESM.docx]

**Supplementary Table. The codes for prediction of the cognitive impairment with the RCFT-copy**

| # %tensorflow_version 2.x  import tensorflow as tf  device_name = tf.test.gpu_device_name()  if device_name != '/device:GPU:0':  raise SystemError('GPU device not found')  print('Found GPU at: {}'.format(device_name))  import matplotlib.pyplot as plt  import numpy as np  import os  import PIL  from glob import glob  from tensorflow import keras  from tensorflow.keras import layers  from tensorflow.keras.models import Sequential  import pathlib  from google.colab import drive  drive.mount('/content/drive/')  data_dir = 'drive/My Drive/Coding/Tensorflow/RCFT_aug/nl_ci'  data_dir = pathlib.Path(data_dir)  image_count = len(list(data_dir.glob('*/*.png')))  print(image_count)  batch_size = 20  img_height = 600  img_width = 600  train_ds = tf.keras.preprocessing.image_dataset_from_directory(  data_dir,  validation_split=0.3,  subset="training",  seed=123,  image_size=(img_height, img_width),  batch_size=batch_size)  val_ds = tf.keras.preprocessing.image_dataset_from_directory(  data_dir,  validation_split=0.3,  subset="validation",  seed=123,  image_size=(img_height, img_width),  batch_size=batch_size)  class_names = train_ds.class_names  print(class_names)  for image_batch, labels_batch in train_ds:  print(image_batch.shape)  print(labels_batch.shape)  break  AUTOTUNE = tf.data.experimental.AUTOTUNE  train_ds = train_ds.cache().shuffle(1000).prefetch(buffer_size=AUTOTUNE)  val_ds = val_ds.cache().prefetch(buffer_size=AUTOTUNE)  normalization_layer = layers.experimental.preprocessing.Rescaling(1. / 255)  normalized_ds = train_ds.map(lambda x, y: (normalization_layer(x), y))  image_batch, labels_batch = next(iter(normalized_ds))  first_image = image_batch[0]  # Notice the pixels values are now in `[0,1]`.  print(np.min(first_image), np.max(first_image))  num_classes = 2  data_augmentation = keras.Sequential(  [  layers.experimental.preprocessing.RandomZoom(0.1),  layers.experimental.preprocessing.RandomRotation(0.012)    ]  )  plt.figure(figsize=(10, 10))  for images, _ in train_ds.take(1):  for i in range(9):  augmented_images = data_augmentation(images)  ax = plt.subplot(3, 3, i + 1)  plt.imshow(augmented_images[0].numpy().astype("uint8"))  plt.axis("off")  model = Sequential([  data_augmentation,  layers.experimental.preprocessing.Rescaling(1./255),  layers.Conv2D(64, 3, padding='same', activation='relu'),  layers.MaxPooling2D(),  layers.Conv2D(64, 3, padding='same', activation='relu'),  layers.MaxPooling2D(),  layers.Conv2D(64, 3, padding='same', activation='relu'),  layers.MaxPooling2D(),  layers.Conv2D(64, 3, padding='same', activation='relu'),  layers.MaxPooling2D(),  layers.Conv2D(128, 3, padding='same', activation='relu'),  layers.MaxPooling2D(),  layers.Dropout(0.4),  layers.Flatten(),  layers.Dense(128, activation='relu'),  layers.Dense(num_classes)  ])  model.compile(optimizer='adam',  loss=tf.keras.losses.SparseCategoricalCrossentropy(from_logits=True),  metrics=['accuracy'])  epochs = 72  with tf.device('/device:GPU:0'):  history = model.fit(  train_ds,  validation_data=val_ds,  epochs=epochs  )  acc = history.history['accuracy']  val_acc = history.history['val_accuracy']  loss = history.history['loss']  val_loss = history.history['val_loss']  epochs_range = range(epochs)  plt.figure(figsize=(8, 8))  plt.subplot(1, 2, 1)  plt.plot(epochs_range, acc, label='Training Accuracy')  plt.plot(epochs_range, val_acc, label='Validation Accuracy')  plt.legend(loc='lower right')  plt.title('Training and Validation Accuracy')  plt.subplot(1, 2, 2)  plt.plot(epochs_range, loss, label='Training Loss')  plt.plot(epochs_range, val_loss, label='Validation Loss')  plt.legend(loc='upper right')  plt.title('Training and Validation Loss')  plt.show() |
| --- |
